# Protracted Morphological Changes in the Corticospinal Tract Within the Cervical Spinal Cord After Intracerebral Hemorrhage in the Right Striatum of Mice

**DOI:** 10.3389/fnins.2020.00506

**Published:** 2020-06-05

**Authors:** Anson Cho Kiu Ng, Min Yao, Stephen Yin Cheng, Jing Li, Jian-Dong Huang, Wutian Wu, Gilberto Ka Kit Leung, Haitao Sun

**Affiliations:** ^1^Department of Surgery, LKS Faculty of Medicine, The University of Hong Kong, Hong Kong, China; ^2^School of Pharmaceutical Sciences, Health Science Centre, Shenzhen University, Shenzhen, China; ^3^The Engineering Technology Research Center of Education Ministry of China, Guangdong Provincial Key Laboratory on Brain Function Repair and Regeneration, Department of Neurosurgery, Zhujiang Hospital, Southern Medical University, Guangzhou, China; ^4^Key Laboratory of Mental Health of the Ministry of Education, Guangdong-Hong Kong-Macao Greater Bay Area Center for Brain Science and Brain-Inspired Intelligence, Southern Medical University, Guangzhou, China; ^5^School of Biomedical Sciences, LKS Faculty of Medicine, The University of Hong Kong, Hong Kong, China; ^6^GHM Institute of CNS Regeneration, Jinan University, Guangzhou, China; ^7^Re-Stem Biotechnology Co., Ltd., Suzhou, China; ^8^Microbiome Medicine Center, Division of Laboratory Medicine, Zhujiang Hospital, Southern Medical University, Guangzhou, China

**Keywords:** intracerebral hemorrhage, hemorrhagic stroke, corticospinal tract integrity, spinal cord changes, collagenase mice model

## Abstract

Intracerebral hemorrhage (ICH) is associated with high morbidity and mortality rates. Currently, there is no promising treatment that improves prognosis significantly. While a thorough investigation of the pathological process within the primary site of injury in the brain has been conducted by the research field, the focus was mainly on gray matter injury, which partly accounted for the failure of discovery of clinically efficacious treatments. It is not until recent years that white matter (WM) injury in the brain after subcortical ICH was examined. As WM tracts form networks between different regions, damage to fibers should impair brain connectivity, resulting in functional impairment. Although WM changes have been demonstrated in the brain after ICH, alterations distant from the initial injury site down in the spinal cord are unclear. This longitudinal study, for the first time, revealed prolonged morphological changes of the contralesional dorsal corticospinal tract (CST) in the spinal cord 5 weeks after experimental ICH in mice by confocal microscopy and transmission electron microscopy, implying that the structural integrity of the CST was compromised extensively after ICH. Given the important role of CST in motor function, future translational studies targeting motor recovery should delineate the treatment effects on CST integrity.

## Introduction

Intracerebral hemorrhage (ICH) damages gray matter, as well as white matter (WM) (Tao et al., 2017; Zuo et al., 2017). White matter tracts, consisting of axons and glial cells, are responsible for signal transduction and ultimately the formation of networks that enable humans to engage in daily activities. Undoubtedly, WM injury contributes to functional disability in cerebrovascular diseases.

Clinical studies have looked into the association between WM tract integrity and functional recovery at different time points and locations after stroke, particularly motor outcomes (Jang et al., 2010). The corticospinal tract (CST) or pyramidal tract is the major descending motor pathway that originates from the motor cortex, passes through the basal ganglia (striatum and globus pallidus) in the internal capsule, and reaches the spinal cord. The structural changes of the CST in the ipsilesional hemisphere down to the contralesional medullary pyramid during acute and chronic phases, as demonstrated in patients by neuroimaging, correlate with motor recovery. This could possibly be used for outcome prediction after ICH (Cho et al., 2007; Yoshioka et al., 2008; Kusano et al., 2009; Jang et al., 2010; Jayaram et al., 2012; Venkatasubramanian et al., 2013). Most cross-sectional and prospective studies used the value of fractional anisotropy as the marker, implying the presence of Wallerian degeneration (WD) of axons after ICH. An animal study provided direct histological evidence of secondary substantia nigra and CST changes in the brain for up to 4 months after experimental ICH (Fan et al., 2013).

The CST, as a source of supraspinal input, modulates activities of the lower motor neurons in the spinal cord. Abnormalities of spinal circuitries have been detected in patients in the chronic phase of stroke, which correlate with motor impairments (Hubli et al., 2012). This means that a unilateral absence of upper motor neuron input due to stroke can lead to downstream functional changes in the spinal cord. While structural changes of CST after stroke have been observed repeatedly in the brain and brainstem, the presence of extension of degeneration into the spinal cord had just been explored. A recent cross-sectional clinical neuroimaging study visualized, for the first time, the microstructural alterations of WM tracts in the brainstem and cervical spinal cord of chronic stroke patients. However, no separate analyses of hemorrhagic and ischemic stroke groups were done (Karbasforoushan et al., 2019). There is a paucity of literature on the changes in spinal cord and ventral roots in rats with or without treatment after ischemic stroke (Wiessner et al., 2003; Liu et al., 2013; Dang et al., 2016). Yet, to the best of our knowledge, there had not been any evaluation of histological changes of CST in the spinal cord after ICH. The present study provided the evidence of prolonged damage to the contralesional dorsal CST in the cervical spinal cords of adult wild-type mice after intrastriatal collagenase injection, a commonly used ICH model.

## Materials and Methods

### Animal

Experimental protocols were approved by the Committee on the Use of Live Animals in Teaching and Research, The University of Hong Kong. Twenty-one adult male C57BL/6N mice, 12–14 weeks old and weighing 25–32 g, were used in this study. Animals were kept for at least 1 week after initial transfer from the laboratory animal unit to minimize the stress associated with novel environment. Animals were kept in a 12-h/12-h light–dark cycle with *ad libitum* access to water and chow. The animal holding areas were under constant monitoring. The temperature was kept at 19 ± 2°C, and the humidity was maintained at 44 ± 2 pw.

### Experimental ICH Model

**FIGURE 1:**
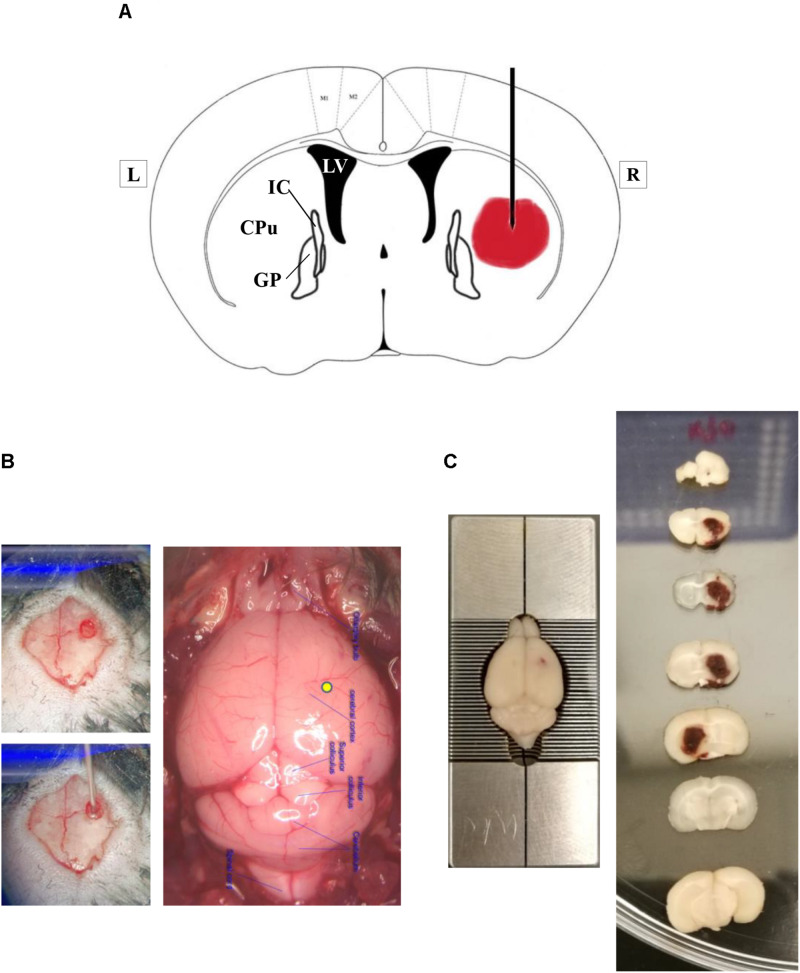
Experimental intracerebral hemorrhage in mice by injection of bacterial type IV collagenase. **(A)** A simplified diagram of a coronal brain section that is 0.2 mm anterior to bregma, illustrating the proposed site of collagenase injection. The lateral ventricle is marked as LV. CPu represents caudate–putamen, a part of the striatum. GP means the globus pallidus. IC is the internal capsule. L, left; R, right. **(B)** Representative photographs showing the location of burr hole on the skull and the injection site on the brain surface (yellow spot). **(C)** Representative photographs of coronal brain sections, obtained at 24 h after ICH. Please note that brain section number 5 was flipped horizontally for better visualization of the hematoma. The hematoma was within the right hemisphere.

The mice were anesthetized with a single intraperitoneal injection of ketamine (100 mg/kg) and xylazine (10 mg/kg). The mice were placed in a stereotactic frame (RWD Life Science Co., Shenzhen, China). Intracerebral hemorrhage was induced by intracranial injection of type IV collagenase (Sigma-Aldrich, St. Louis, MI, United States). The right corpus striatum was the target region (Figure 1). In summary, a burr hole of 0.6 mm in diameter, which was 0.2 mm anterior to the bregma and 2.0 mm lateral to the midline, was drilled. A 30-gauge needle was inserted into the right striatum with its tip 3.5 mm below the dural surface. Intracerebral hemorrhage was induced by a slow injection of 0.04 U type IV collagenase in 0.5 μL saline into the right striatum at a rate of 0.15 μL/min.

### Experimental Groups

The mice were randomly assigned to one of the two groups: sham operation group with needle insertion only (*n* = 6) and ICH group with the injection of type IV collagenase (*n* = 15). The mice in the sham group were randomly divided into two time points: week 1 (W1) and week 4 (W4) after sham operation (*n* = 3 each group). The mice in the ICH group were randomly divided into five time points: week 1 (W1), week 2 (W2), week 3 (W3), week 4 (W4), and week 5 (W5) postoperation (*n* = 3 each group).

### Tissue Preparation for Transmission Electron Microscopy and Histology

At W1, W2, W3, W4, and W5 after ICH induction as well as at W1 and W4 after sham operation, 3 mice per group were deeply anesthetized by pentobarbital sodium (200 mg/kg, intraperitoneally); the mice were transcardially perfused with 1 × phosphate-buffered saline (PBS, pH 7.4) and 4% paraformaldehyde in 0.1 M PBS. The cervical segment of the spinal cord of each mouse was collected and fixed overnight in 2.5% glutaraldehyde in 0.1 M PBS. The samples were postfixed in 1% osmium tetroxide. After progressive dehydration in ethanol, samples were embedded in epoxy resin and sectioned with an ultramicrotome (UC6; Leica, Wetzlar, Germany) for toluidine blue staining and transmission electron microscopic analysis.

### Confocal Microscopic and Transmission Electron Microscopic Analysis

Preparation of slides for microscopic analysis was done by methods described previously with slight modification (Dang et al., 2016). Semithin (230 nm) and ultrathin (100 nm) horizontal cross-sections were used for confocal microscopic and transmission electron microscopic analyses, respectively, to delineate the phenotypic changes of the cervical segment of the CST. Semithin sections were stained with toluidine blue, dehydrated with ethanol, and examined under a confocal microscope (LSM 780; Carl Zeiss, Oberkochen, Germany) (63×, oil immersion) using bright field. Ultrathin sections were stained in saturated uranyl acetate and lead citrate. These sections were then viewed and photographed under a transmission electron microscope (CM100 Transmission Electron Microscope; Philips, Eindhoven, Netherlands) (1,200 × 3,900×). Blinding was not done because of the obvious alternations seen on the tissue sections.

### Semiquantification of Degenerative Changes in CST

Quantification was done by methods described previously with slight modification (Mi et al., 2007). Corticospinal tract degeneration led to the appearance of an obviously darkened region in the contralesional CST. One large field (120 × 40 μm^2^, located along the line separating two CST and 40 μm away from a straight line parallel to field that met the tip of each CST zone, 63 × oil immersion) within each contralesional and ipsilesional CST on horizontal toluidine blue–stained spinal cord sections chosen randomly from the fifth cervical segment of each animal was used for measuring a total area containing intact myelin sheaths, degenerated axons, and myelin sheaths using ImageJ software^1^. To estimate the magnitude of changes in the contralesional CST, a ratio of the total area of the contralesional CST field to that of the ipsilesional CST field was used. The contralesional and ipsilesional fields were located in the same sections to minimize differences due to intersection variation except for the comparison of ipsilesional CST fields between sham and ICH groups.

### Statistical Analysis

GraphPad Prism (version 8) software^2^ was used for statistical analysis. Data were shown as means ± standard error of mean. Statistical significance in comparing two groups was tested with the unpaired Student *t* test (with Welch’s correction or Mann-Whitney modification). One-way analysis of variance followed by Tukey multiple-comparisons test was used to compare means of three or more groups. *p* < 0.05 was set as the cutoff for statistical significance.

## Results

### Lasting Changes in the Overall Pathology of the Dorsal CSTs After ICH

**FIGURE 2:**
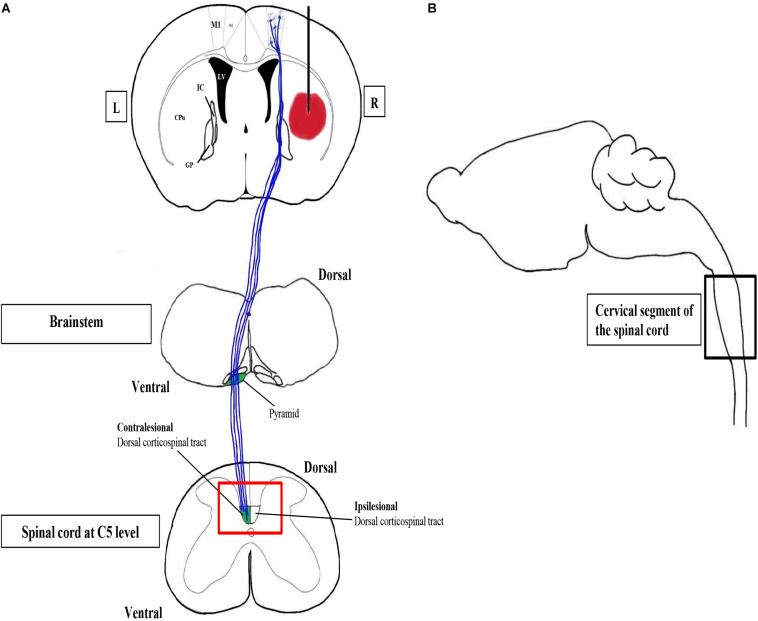
Confocal and transmission electron microscopy to study CST changes after ICH. The corticospinal tract (CST) is the major system for skilled voluntary movement in human and motor functions in rodents. Motor recovery after stroke critically depends on CST integrity. The CST is the only descending pathway in which some axons form synapses with spinal motor neurons directly. M1 is the primary motor cortex containing pyramidal neurons, the axons of which constitute the CST. The CST, which is highlighted in blue, passes through the internal capsule (IC) to the pyramids of the brainstem, where the majority of CST fibers decussate to the contralateral side. **(A)** A simplified coronal section of the brain with the hematoma in the right striatum, together with schematic diagrams of the horizontal sections of the brainstem and the spinal cord, respectively. The red box is the region of interest, the CST zone at C5 level, of our study. L, left; R, right. **(B)** A simplified parasagittal section of the brain and spinal cord.

**FIGURE 3:**
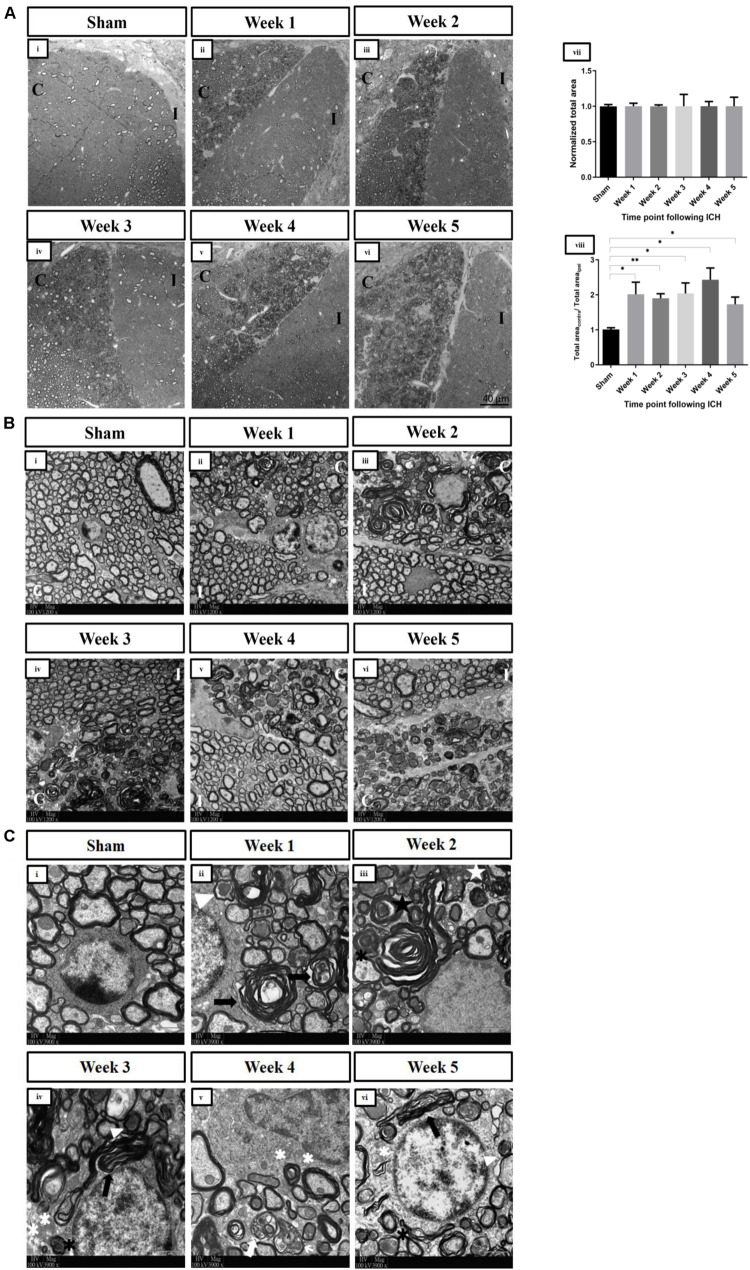
**(A)** Changes in the overall pathology of the dorsal corticospinal tracts in spinal cord at the C5 level post-ICH in adult mice detected by confocal microscopy. Representative toluidine blue–stained semithin horizontal sections of dorsal corticospinal tracts at the C5 spinal cord level demonstrating abnormalities particularly in the contralesional corticospinal tract under bright field (63×, oil immersion) at (ii) W1 post-ICH, (iii) W2 post-ICH, (iv) W3 post-ICH, (v) W4 post-ICH, and (vi) W5 post-ICH compared to (i) sham group at W1. (vii) The normalized areas of ipsilesional CSTs of the sham and ICH groups, demonstrating that there were no significant changes in the ipsilesional CSTs after ICH (*p* > 0.05) (viii) The ratios of total areas of contralesional CST fields to that of ipsilesional CST fields, showing that abnormal axons and myelin sheaths were present significantly in the contralesional dorsal corticospinal tract in the spinal cord after ICH for weeks (*p < 0.05, **p < 0.01; compared to sham group). **(B)** Axonal degeneration and myelin sheath abnormalities of the contralesional dorsal corticospinal tract in spinal cord at the C5 level post-ICH in adult mice detected by transmission electron microscopy. Low-magnification electron micrographs (1,200×) of bilateral dorsal corticospinal tracts. (i) Representative microphotograph showing normal axons with intact myelin sheaths in the sham group at W1. A loss of homogeneity of axons was seen at (ii) W1 post-ICH, (iii) W2 post-ICH, (iv) W3 post-ICH, (v) W4 post-ICH, and (vi) W5 post-ICH. **(C)** Examples of abnormalities of axons and myelin sheaths of the contralesional dorsal corticospinal tract in the spinal cord at the C5 level post-ICH in adult mice detected by transmission electron microscopy. High-magnification electron micrographs (3,900×) of contralesional corticospinal tract. (i) Normal axons with intact myelin sheaths in the sham group at W1. (ii) Dark degeneration of axons (white arrowhead) and abnormal myelin sheaths (black arrows) at W1 post-ICH. (iii) Accumulation of membranous debris within the axoplasm (white star), degenerated axon wrapped by loose myelin sheath lamellae (black asterisks), and infolding of myelin sheath (black star) at W2 post-ICH. (iv) Demyelinated axons (white asterisks) at W3 post-ICH. (v) Debris from the myelin sheath surrounding the shrunken axons (white arrows) at W4 post-ICH. (vi) The pathologies persisted at W5 post-ICH. C, contralesional CST; I, ipsilesional CST. *n* = 3 for each group. The statistical difference between two groups was tested with the unpaired Student *t* test (with Welch’s correction or Mann-Whitney modification). One-way analysis of variance followed by Tukey multiple-comparisons test was used to compare means of three of more groups.

After the induction of ICH in the right striatum, visualization of the dorsal CSTs in the fifth segment of the cervical spinal cord by confocal microscopy revealed pathological changes in the contralesional CST (Figure 2). Bilateral CSTs were normal after the sham operation at W1 [Figure 3A(i)] and W4 (data not shown). After ICH, there was an absence of degenerative changes in the ipsilesional CST. However, the injury to the right hemisphere led to obvious derangement of WM on the left side of the cervical spinal cord, which persisted for at least 5 weeks after ICH [Figure 3A(ii–vi)]. The total areas were similar between the contralesional and ipsilesional CSTs in the sham group, giving a ratio of approximately 1 [Figure 3A(viii)]. The normalized total areas of the ipsilesional CST fields of the sham group were not statistically different from that of the ipsilesional CST fields of ICH groups at all time points, indicating that there were no significant degenerative changes in the ipsilesional CST after ICH [Figure 3A(vii)]. The ratios of the total area of the contrlesional CST fields to that of the ipsilesional CST fields of the all ICH groups were significantly higher than those of the sham group, implying that the degenerative changes are significant in the contralesional CST. There was no significant difference in the ratios between time points after ICH [Figure 3A(viii)].

### Axonal Degeneration and Myelin Abnormalities of the Contralesional Dorsal CST in the Spinal Cord After ICH

Electron microscopy was used to visualize different abnormalities of the WM after ICH at higher magnifications. Normal axons and myelin sheaths were seen in the sham group [Figure 3B(i)] and the ipsilesional CST at C5 level [Figure 3B(ii–vi)]. In contrast, there was an abnormal heterogeneity of shape and size of axons within the contralesional CST after ICH [Figure 3B(ii–vi)]. An array of pathologies was observed in the contralesional CST [Figure 3C(ii–vi)], including dark degeneration of axons, demyelination, membranous debris deposition in the axoplasm, aberrant myelin wrapping, and infolding of myelin sheaths. These changes, which represent WD of axons, were apparent even 5 weeks after ICH.

## Discussion

This is the first illustration of the longitudinal pathological alternations of the contralesional CST in the cervical portion of the spinal cord after unilateral striatal hemorrhage in adult mice. White matter degeneration that lasted for at least 5 weeks after ICH, that is, even during the chronic phase after stroke, was detected. The changes included axonal degeneration, demyelination of axons, and myelin sheath abnormalities. Given the proximity of the internal capsule, which contains fibers of the CST, to the striatum, part of the CST might have sustained direct damage by the hematoma and perihematomal edema. This has been demonstrated in some clinical neuroimaging studies (Cho et al., 2007; Yoshioka et al., 2008). However, the observed pathological changes in such distant region could also indicate secondary degeneration caused by factors other than the primary injury. In fact, one article showed prolonged ipsilesional CST changes in the brain without direct contact with the unilateral ICH in rats (Fan et al., 2013). This assumption is also supported by a clinical study that specifically detected WD of CST in areas with the absence of hemorrhage (Venkatasubramanian et al., 2013). Moreover, several research groups investigated the effect of ICH on neurons in distant brain regions, such as the hippocampus and substantia nigra in recent years (Yang et al., 2015, 2018a,b; Lei et al., 2016). Although these areas are not close to the hematoma, they sustain damage. Hence, by inference, a combination of primary damage and secondary degeneration of WM possibly led to the phenotype seen in this study.

Wallerian degeneration, local inflammation, and oxidative stress are possible mechanisms that perpetuate secondary degeneration of CST fibers. The cell bodies of CST are located in the cortex. Hence, a subcortical injury to the CST disconnects the axons from the cell bodies. This triggers myelin degradation and derangement of axonal structures, together with inflammatory cell infiltration caudal to the site of injury (Tennant, 2014). Wallerian degeneration may further damage initially spared axons (Weishaupt et al., 2010). Axonal degeneration recruits microglia and possibly peripheral immune cells after the breakage of blood–brain barrier, which partake in debris clearance and inflammation. The release of proinflammatory cytokines, cytotoxic proteins, and reactive oxygen species by these cells exacerbates injury to the WM tracts locally, forming a vicious cycle of axonal degeneration, demyelination, and inflammation. However, the functional significance of local secondary degeneration is not yet determined. On the other hand, it has been shown by studies on ischemic stroke that axonal regeneration and remyelination occur within the spinal cord (Sist et al., 2014; Kaiser et al., 2019). Hence, the data shown by this study can imply a balance between degenerative and regenerative processes, resulting in an incomplete recovery of CST after ICH.

In contrast to the bilateral CST alterations in the hemispheres after ICH (Fan et al., 2013), abnormalities were observed only in the contralesional CST in the cervical spinal cord. The reason could be that the transient changes in WM diffusivity, as measured by diffusion tensor imaging (DTI), of the contralesional CST in the brain during the first few days after ICH were insufficient to induce secondary degeneration in the chronic phase. Yet, the mechanisms driving the temporary change and recovery of contralesional CST were not investigated.

The discrepancy between the CST changes in the brain and in the spinal cord points out one of the roles the spinal cord could play in ICH-related research. It may be less useful to solely focus on spinal cord changes as the primary injury site is in the brain. Changes in remote areas should occur in a relatively delayed manner and possibly reflect the severity of injury originated from the brain. Therefore, the spinal cord phenotype could be seen as an indicator of the residual integrity of long WM tracts after stroke alternatively. A growing body of clinical evidence highlights the importance of structural and functional CST integrity in motor recovery after ischemic stroke (Byblow et al., 2015; Bigourdan et al., 2016). Unlike the irreversible primary damage to WM by mechanical stress and hemorrhage in the brain, the ongoing CST degeneration and demyelination are amenable to intervention. Although the minimal residual integrity may not contribute to recovery after severe ICH, that of mildly to moderately injured CST should help. Corticospinal tract integrity could either be maintained via minimizing degeneration, or enhanced by promoting plasticity, as the central nervous system has a functional albeit limited regenerative capacity after injury (Sist et al., 2014; Tennant, 2014). Future studies of our laboratory will focus on treatments that could protect the CST integrity after ICH.

## Conclusion

This study demonstrated for the first time in an animal model that WD of CST continued for no less than 5 weeks after ICH. Such degeneration represented the long-lasting effect of ICH on distant regions connected to or in the vicinity of the primary injury site. As the CST is involved in mediating motor functions in both animals and human, interventions that protect or improve CST integrity should aid recovery.

This study has several limitations. We did not correlate the severity of CST changes with neurological deficit. Future studies will measure the structural and functional integrity of the CST and the degree of neurological deficit in the presence of a treatment. Neuroimaging techniques such as DTI could be used to indicate structural integrity of WM tracts in the spinal cord, but it is difficult to perform in mice because of the small size of the spinal cord and insufficient spatial resolution of current technology (Vedantam et al., 2014). We are in search of suitable methods to reliably demonstrate different aspects of the integrity of the CST after ICH. In the present study, we did not examine the loss of motor neurons in the cervical spinal cord after ICH. However, a previous study revealed significant losses of neurons in the ventral horns of the cervical and lumbar enlargements after experimental ischemic stroke (Dang et al., 2016). Therefore, similar pathological changes are likely to be observed after ICH. In addition, few clinical studies elicited the dysfunction of spinal neurons and spinal reflex after ischemic stroke (Van Kuijk et al., 2007; Hubli et al., 2012). All in all, the evidence suggests the possibility of spinal motor neuron loss after ICH. Moreover, we hope to investigate the changes in other WM tracts affected by striatal hemorrhage and the associations with ICH outcomes in the future.

## Data Availability Statement

All datasets generated for this study are included in the article/supplementary material.

## Ethics Statement

The animal study was reviewed and approved by the Committee on the Use of Live Animals in Teaching and Research, The University of Hong Kong.

## Author Contributions

The work presented here was carried out in collaboration among all authors. HS, WW, GL, and J-DH conceived the research. GL and HS designed the research. AN, HS, MY, and SC performed the research. AN, HS, MY, WW, and JL analyzed the data. AN, HS, and GL wrote the manuscript. All authors read, commented on, and approved this manuscript.

## Conflict of Interest

WW was part-time employed by the company Re-Stem Biotechnology Co., Ltd., Suzhou. The remaining authors declare that the research was conducted in the absence of any commercial or financial relationships that could be construed as a potential conflict of interest.

## Footnotes

1 Rasband, W. S., ImageJ, U. S. National Institutes of Health, Bethesda, Maryland, United States, https://imagej.nih.gov/ij/, 1997–2018

2 GraphPad Software, San Diego, California, United States, www.graphpad.com
